# Effect of Rumen-Protected Methionine on Metabolic Profile of Liver, Muscle and Blood Serum Samples of Growing German Simmental Bulls Fed Protein-Reduced Diets

**DOI:** 10.3390/metabo13080946

**Published:** 2023-08-15

**Authors:** Vivienne Inhuber, Wilhelm Windisch, Karin Kleigrewe, Chen Meng, Benedikt Bächler, Michael Gigl, Julia Steinhoff-Wagner, Thomas Ettle

**Affiliations:** 1 Chair of Animal Nutrition and Metabolism, Technical University of Munich, Liesel-Beckmann-Strasse 2, 85354 Freising-Weihenstephan, Germany; vivienne.inhuber@tum.de (V.I.); wilhelm.windisch@tum.de (W.W.);; 2 Bavarian Center for Biomolecular Mass Spectrometry, TUM School of Life Sciences, Gregor-Mendel-Strasse 4, 85354 Freising, Germany; karin.kleigrewe@tum.de (K.K.);; 3 Bavarian State Research Center, Institute for Animal Nutrition and Feed Management, Prof.-Duerrwaechter-Platz 3, 85586 Poing, Germany

**Keywords:** ruminants, amino acids, anti-oxidants, nutrient partitioning, metabolomics, ideal protein concept

## Abstract

This study aimed to determine the metabolic response of growing German Simmental bulls fed rations low in crude protein (CP) supplemented with rumen-protected methionine (RPMET). In total, 69 bulls (on average 238 ± 11 days of age at start and 367 ± 25 kg of bodyweight) were assigned to three dietary treatments (*n* = 23/group): Positive control (CON; 13.7% CP; 2.11 g methionine/kg DM), negative control deficient in CP (RED; 9.04% CP; 1.56 g methionine/kg DM) and crude protein-deficient ration supplemented with RPMET (RED+RPMET; 9.04% CP; 2.54 g methionine/kg DM). At slaughter, samples of liver, muscle and blood serum were taken and underwent subsequent metabolomics profiling using a UHPLC-QTOF-MS system. A total of 6540 features could be detected. Twenty metabolites in the liver, five metabolites in muscle and thirty metabolites in blood serum were affected (*p <* 0.05) due to dietary treatments. In total, six metabolites could be reliably annotated and were thus subjected to subsequent univariate analysis. Reduction in dietary CP had minimal effect on metabolite abundance in target tissues of both RED and RED+RPMET bulls as compared to CON bulls. The addition of RPMET altered the hepatic anti-oxidant status in RED+RPMET bulls compared to both RED and CON bulls. Results exemplify nutrient partitioning in growing German Simmental bulls: bulls set maintenance as the prevailing metabolic priority (homeostasis) and nutrient trafficking as the second priority, which was directed toward special metabolic functions, such as anti-oxidant pathways.

## 1. Introduction

Beef supply chains have gained significant attention in the global discussions on livestock production. This public opinion is mainly derived from the fact that beef cattle have lower feed-to-food transformation efficiency as compared to both poultry and swine [1], meanwhile excluding other specific attributes of beef cattle to explain their importance for a bio-circular economy [2,3]. However, in recent decades, nutritional strategies that have focused on increasing the efficiency of livestock have been well established, including the ‘ideal protein concept’ in monogastric nutrition. The ‘ideal protein’ denotes a dietary protein that does not under- or oversupply intestinal digestible amino acids [4] and thereby ensures an efficient feed-to-food protein turnover. This concept allows for a reduction in dietary crude protein due to the supplementation of single amino acids. The successful application of this concept, however, requires precise knowledge on the metabolic role and requirements of single performance-limiting amino acids [5].

This nutritional concept cannot be simply transferred to beef and dairy nutrition, since the fore stomach system synthesizes its own protein in the rumen (microbial protein). Thus, there is an incongruence between ingested and absorbed amino acid patterns, which impedes the determination of performance-limiting amino acids [6].

Nevertheless, numerous studies imply that lysine and methionine might be the first-limiting amino acids in growing ruminants [6], more specifically when microbial protein derived from the rumen is the major supplier of amino acids at the small intestine [7].

Methionine is a sulfurized amino acid, which enters the one-carbon metabolism in its active form S-adenosylmethionine. As such, it donates its methyl group to an acceptor, thereby producing S-adenosylhomocysteine, which can be then hydrolyzed to homocysteine. This trans-methylation process, which is restricted to the liver, kidney, intestine and pancreas, is the first step of numerous subsequent metabolic pathways. It is followed by the trans-sulfuration pathway, which converts homocysteine into cysteine. Cysteine, in turn, serves as the intermediate metabolite for glutathione synthesis, an important antioxidant, or is oxidized into cystine, for instance. Other metabolic fates of cysteine are, e.g., the synthesis of taurine. Taurine, in turn, has several metabolic functions, such as antioxidant activity [8,9,10].

Protecting dietary methionine from ruminal degradation increases its metabolic availability in ruminants [11]. This has recently been demonstrated in a study [6], where growing German Simmental bulls received either a control diet (CON) or a diet low in crude protein (RED) supplemented with rumen-protected methionine (RPMET; RED+RPMET; 0.16% of DM Smartamine M^®^, Adisseo, France). Reduction in dietary crude protein led to a decrease (*p <* 0.05) in growth performance over the whole experiment in both RED and RED+RPMET as compared to the CON group. The addition of RPMET did not relieve the RED+RPMET bulls from growth limitations, suggesting that under those feeding conditions, methionine was not firstly limiting for growth. However, the supplementation of RPMET led to an increase (*p <* 0.01) in serum methionine concentrations in RED+RPMET as compared to RED bulls (43.4 vs. 35.0 μmol/L) and hence, increased its metabolic availability [6]. Based on this background, the objective of this study was to evaluate the metabolic response of growing German Simmental bulls to diets low in crude protein with supplemented RPMET.

## 2. Materials and Methods

### 2.1. Feeding Experiment

The feeding experiment was conducted at the Bavarian State Research Center for Agriculture (LfL, Grub, Germany). All experimental procedures followed the guidelines of the German law under the German State and Directive 2010/63/EU of the Parliament and of the Council of 22 September 2010 on the protection of animals used for scientific purposes. Slaughter of the animals was conducted in accordance with the German law of animal protection of the German State and Council Regulation (EC) No. 1099/2009 of 24 September 2009 on the protection of animals at the time of killing.

#### 2.1.1. Animals and Dietary Treatments

A feeding trial was conducted to harvest liver and muscle tissue and serum samples at the slaughter of the bulls. 

In brief, a total of 69 growing fattening German Simmental bulls (on average 238 ± 11 days of age at beginning of the experiment; 367 ± 25 kg initial bodyweight) were allotted to a control group (CON) and two dietary treatment groups (*n* = 23/group). Bulls were weighed at the beginning and immediately before slaughter as well as in regular intervals during the experimental period. The CON ration provided adequate metabolizable energy (ME; 12.3 MJ/kg total mixed ration), crude protein (13.7%) and utilizable crude protein (15.7%), referring to the current national recommendations of German Simmental bull feeding [12]. Both treatment groups (RED and RED+RPMET) contained less crude protein (both 9.04%) and utilizable crude protein (both 14.9%), but were supplemented with rumen-protected lysine to reach CON diet levels. The diets RED and RED+RPMET only differed in their concentration of digestible methionine (2.2 g/kg dry matter vs. 3.2 g/kg dry matter, respectively) due to supplementation of RPMET (0.16% of dry matter; Smartamine M^®^, Adisseo SAS, Antony, France). The feeding trial lasted for 105 days on average. 

Details on the feeding experiment were previously described [6].

#### 2.1.2. Slaughter and Sample Collection

At the end of the feeding experiment, bulls were slaughtered over a time period of eight d and bulls from all three treatments were slaughtered on each date. At slaughter, liver (3 cm^2^ of the left lobe of the liver) and muscle (*Musculus masseter*) tissue samples of each bull were removed and directly frozen in a liquid nitrogen container to minimize metabolic degradation. Samples were stored in 1.5 mL cryotubes at −80 °C until assayed. Additionally, blood samples were collected in Vacuette tubes (VACUETTE TUBE 4 mL CAT Serum Clot Activator, Greiner Bio-One International GmbH, Kremsmünster, Austria) during exsanguination. Vacuette tubes were inverted and centrifuged (2000× *g* 10 min, at 4 °C) and the serum samples were transferred to 1.5 mL cryotubes and then stored at −80 °C until further analysis.

### 2.2. Sample Preparation and Metabolomics Analysis

Muscle, liver and serum samples were thawed at 4 °C. Subsequently, 10 mg of each tissue (muscle and liver) sample were transferred into 2 mL FastDNA Lysing Matrix A tubes containing lysing matrix A for soft animal tissues (MP Biomedicals, Solon, OH, USA). After adding 1 mL of an extraction solvent (70% methanol, 30% water), samples were homogenized using FastPrep-24TM Classic (MP Biomedicals, Irvine, CA, USA; 30 s, 5500 RPMET, 4 °C, 2 times) and incubated on ice (30 sec after each spin). Samples were then centrifuged (10,000× *g*, 5 min at 4 °C) and the clear supernatants were transferred to 1.5 mL glass autosampler vials. Blood serum samples (200 μL) were diluted in the same extraction solvent used for tissue samples (1 mL). 

Untargeted metabolomics analyses were carried out at the Bavarian Center for Biomolecular Mass Spectrometry (BayBioMS, Technical University of Munich, Freising, Germany). The untargeted analysis was performed using a Nexera UHPLC system (Shimadzu), which was coupled to a Q-TOF mass spectrometer (TripleTOF 6600, AB Sciex, Toronto, Ont., Canada) using the information-dependent acquisition (IDA) mode. A UPLC BEH Amide 2.1 × 100, 1.7 µm analytic column (Waters Corp., Milford, MA, USA) with a 400 µL/min flow rate was used for separation of the biological samples. The mobile phase consisted of eluent A (5 mM ammonium acetate in water) and eluent B (5 mM ammonium acetate in acetonitrile/water (95/5, *v*/*v*)) and was performed with the following elution gradient: 100% B from 0 to 1.5 min, 60% B at 8 min and 20% B at 10 min to 11.5 min and 100% B at 12 to 15 min. A volume of 10 µL per sample was injected. The autosampler was cooled to 10 °C and the column oven was heated to 40 °C. A quality control (QC) sample was run after every ten samples (analyzed in randomized order). It consisted of a pool of all injected samples. MS settings in the positive mode were as follows: gas 1, 55; gas 2, 65; curtain gas, 35; temperature, 500 °C; ion spray voltage, 5500; declustering potential, 80. The mass range of the TOF MS and MS/MS scans were 50–2000 m/z. The collision energy was ramped from 15 to 55 V. MS settings in the negative mode were set as follows: gas 1, 55; gas 2, 65; cur, 35; temperature, 500 °C; ion spray voltage, −4500; declustering potential, −80. The mass range of the TOF MS and MS/MS scans was 50–2000 m/z. The collision energy was ramped from −15 to −55 V.

### 2.3. Statistical Data Analysis and Bioinformatics

MS raw data files were converted to mzXML via the “msconvert” from ProteoWizard [13]. Data processing and feature identification were conducted with the Bioconductor/R package XCMS [14]. In detail, peaks were identified with the matched filter algorithm (full width at half maximum set to 7.5 s). Grouping of the peaks was based on the “peak density” method [14]. Feature abundance was represented by the integrated area under the peaks. Peak groups presented in most samples were used to adjust the retention time. The MS1 (exact mass of the precursor ion) and MS2 (fragmentation pattern of precursor ion) of the measured features were compared to the records in the Human Metabolome Database (HMDB) [15] and published MS2 spectra compiled by MSDIAL [16] to annotate possible metabolites for the identification of features (referring to MS1 and MS2, respectively). Additionally, in-house annotation database was taken into consideration for feature annotation. Potential batch effects were controlled and removed according to QC samples. Features were categorized into four categories depending on the quality of annotation: One means only the feature mass is matched with the metabolite candidate in the database, and no MS2 information is used. Zero means the MS2 spectra matching suggests the annotation is likely incorrect. Two means the partial match of MS2 spectra (at least one peak match). Four means high similarity between measured MS2 spectra and database MS2 spectra. The associated untargeted metabolomics data are made available on the MassIVE repository under the ID MassIVE MSV000092367: https://massive.ucsd.edu/ProteoSAFe/static/massive.jsp, last accessed on 16 July 2023. 

Univariate analysis was performed using SAS (SAS 9.4, SAS Institute, Cary, NC, USA). Treatment group means of significantly regulated metabolites, which were obtained from XCMS analyses, were tested via normal distribution (Shapiro–Wilk’s test) and then analyzed using a general linear model (GLM) with treatment as fixed effect and a Student–Newman–Keuls (SNK) post-hoc test according to the SAS user’s guide [17]. The *p*-value of the GLM represents the statistical significance of the GLM model, whereas the *p*-values of the linear contrasts I and II indicate differences in response variables due to reduction in dietary crude protein and addition of RPMET (CON vs. RED+RPMET) and due to the addition of RPMET (RED vs. RED+RPMET). The SEM represents the standard error of the mean over the whole GLM. Statistical significance was declared at *p* ≤ 0.05.

## 3. Results

### 3.1. Zootechnical Results

Zootechnical results on the feeding experiment were previously described [6]. In brief, dietary treatments had a significant effect on performance parameters. Bulls from both the RED and RED+RPMET groups had a lower (*p <* 0.05) dry matter intake (8.49 kg/day and 8.27 kg/day, respectively) as compared to the standard group (9.43 kg/day in CON) over the whole duration of the experiment. This led to a reduction (*p <* 0.01) in digestible protein, metabolizable energy and nutrient intake in both RED and RED+RPMET groups as compared to the CON group. Consequently, growth rates were lower in RED and RED+RPMET bulls (*p <* 0.01) than in CON bulls. Considering the lower growth rates in both RED and RED+RPMET in a retrospective view, digestible protein supply in both RED and RED+RPMET met their requirements at 133 and 138%, respectively. Due to the specific addition of RPMET in RED+RPMET, these bulls had a higher (*p <* 0.01) intake of pre-cecal digestible methionine (26.2 g/day) as compared to RED bulls (18.8 g/day). Again, considering the lower growth rates in a retrospective view, methionine intake matched their daily requirements at 146% and 101%, respectively.

### 3.2. Relative Quantification and Identification of Compounds in Liver and Muscle Tissue and Blood Serum Samples

In total, 930 and 527 features were identified in positive (P) and negative (N) ionization modes, respectively, in liver tissue. A total of 1122 and 747 features were identified in P and N ionization modes, respectively, in muscle tissue, and 1835 and 1379 features were identified in blood serum samples, respectively (Table 1).

**Figure 1:**
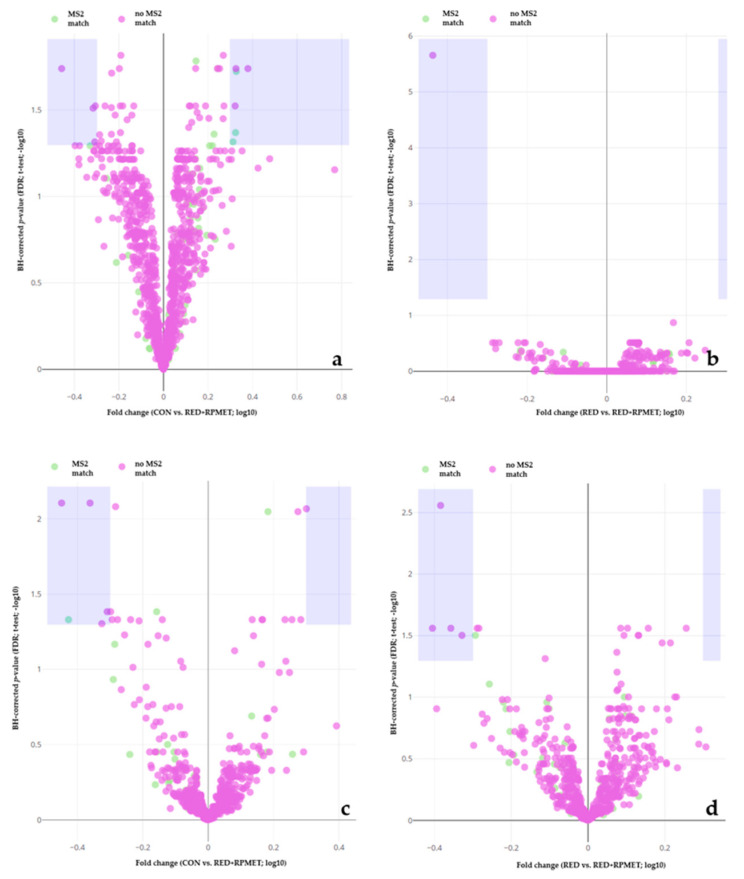
Volcano plots of liver metabolites. The comparison of CON vs. RED+RPMET is depicted in either the P detection mode (**a**) or the N detection mode (**c**) and the comparison of RED vs. RED+RPMET in either the P detection mode (**b**) or the N detection mode (**d**). Y-axis depicts Benjamini–Hochberg (BH)-adjusted *p*-value’s false discovery rate (FDR). X-axis depicts the fold change between the treatment groups with CON = control diet according to requirements, RED = reduced in crude protein and RED+RPMET = reduced in crude protein + addition of RPMET (0.16% DM).

**Figure 2:**
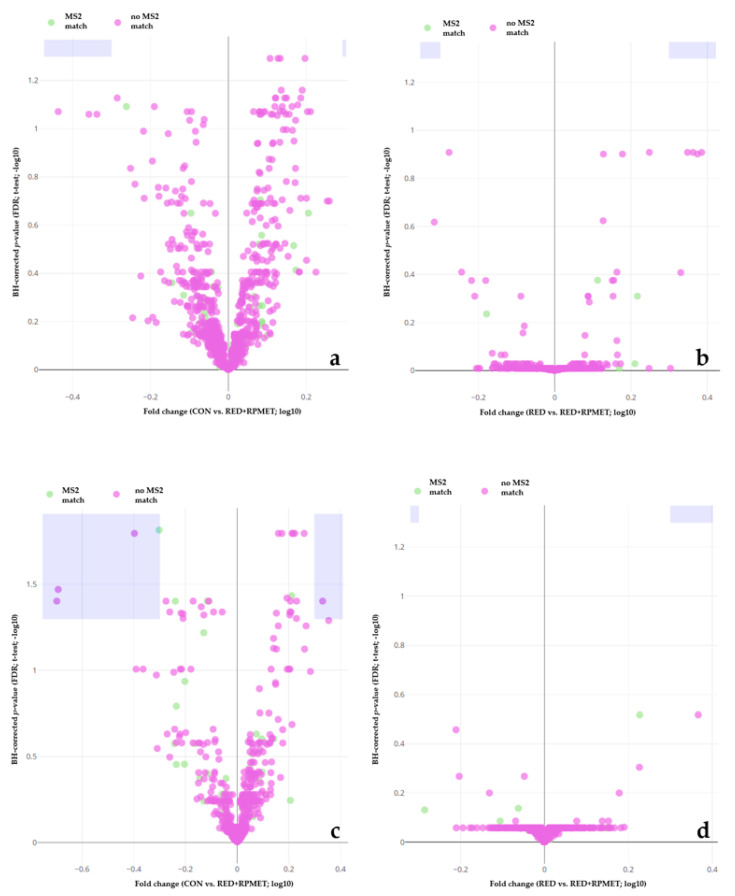
Volcano plots of muscle metabolites. The comparison of CON vs. RED+RPMET is depicted in either the P detection mode (**a**) or the N detection mode (**c**) and the comparison of RED vs. RED+RPMET in either the P detection mode (**b**) or the N detection mode (**d**). Y-axis depicts Benjamini–Hochberg (BH)-adjusted *p*-value’s false discovery rate (FDR). X-axis depicts the fold change between the treatment groups with CON = control diet according to requirements, RED = reduced in crude protein and RED+RPMET = reduced in crude protein + addition of RPMET (0.16% DM).

**Figure 3:**
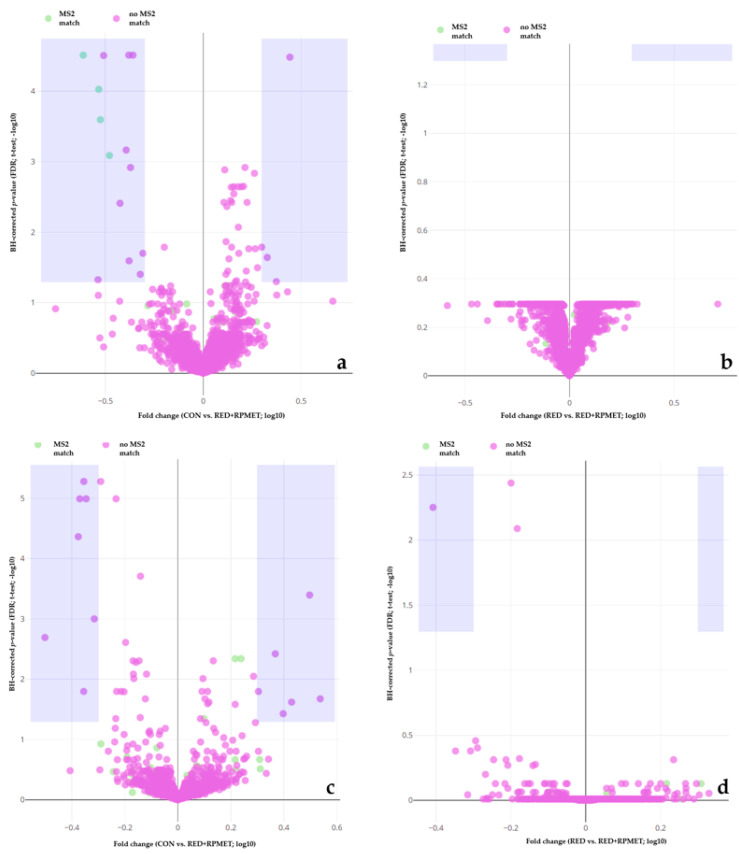
Volcano plots of blood serum metabolites. The comparison of CON vs. RED+RPMET is depicted in either the P detection mode (**a**) or the N detection mode (**c**) and the comparison of RED vs. RED+RPMET in either the P detection mode (**b**) or the N detection mode (**d**). Y-axis depicts Benjamini–Hochberg (BH)-adjusted *p*-value’s false discovery rate (FDR). X-axis depicts the fold change between the treatment groups with CON = control diet according to requirements, RED = reduced in crude protein and RED+RPMET = reduced in crude protein + addition of RPMET (0.16% DM).

Figure 1, Figure 2 and Figure 3 visualize liver, muscle and blood serum metabolites that were detected during metabolomics analysis. Violet areas in the volcano plots display significantly regulated metabolites. The significance level was set at 0.05 for the false discovery rate (FDR) of Benjamini–Hochberg (BH)-corrected *p*-value. Features depicted in ‘green’ show a match with MS2 spectra, whereas features in ‘violet’ lack a respective match. Each figure shows four volcano plots. In all figures (Figure 1, Figure 2 and Figure 3), volcano plots 1 show the comparison between CON and RED+RPMET and volcano plots 2 visualize the comparison between RED and RED+RPMET, in P (a) and N (b) detection (ionization) mode each.

Overall, there were 20 significantly regulated metabolites in liver tissue, from which 15 were significantly different between CON and RED+RPMET (5 detected in N mode and 10 detected in P mode) and five between RED and RED+RPMET (four detected in N mode and one detected in P mode), respectively (Figure 1).

In muscle tissue, five metabolites were found to be statistically different between CON and RED+RPMET (all detected in N mode) (Figure 2).

In total, 30 serum metabolites were significantly regulated, 29 between CON and RED+RPMET (13 detected in N mode and 16 detected in P mode) and one between RED and RED+RPMET (detected in N mode).

### 3.3. Univariate Analysis of Annotated Metabolites

Table 2 shows the results of the univariate analysis of significantly regulated metabolites that could be annotated reliably. This means that the MS2 spectra of these metabolites showed a high similarity with the MS2 spectra of the databases used for analysis (compare Section 2.3). Reduction in dietary crude protein (CON vs. RED) led to a decrease (*p <* 0.01) in hepatic carnosine (5.09 vs. 4.79), cystine (4.65 vs. 4.18) and taurocholic acid (4.69 vs. 4.31) and blood serum pyrrolidonecarboxylic acid (4.61 in CON vs. 3.97 in RED) concentrations. Additionally, the MS1 and MS2 spectra indicated the presence of L-leucine/L-isoleucine/norleucine with (*p <* 0.01) between CON and RED (4.37 vs. 4.13). Based on the retention time and the fragmentation pattern, we cannot differentiate between these amino acids and therefore combine them. The addition of RPMET (RED+RPMET) did not affect metabolite concentrations as compared to RED, with the exception of taurocholic acid (4.53 vs. 4.31, *p* Lin. Contrast II = 0.01) and cysteine glutathione disulfide (4.96 in RED+RPMET vs. 4.63 in RED; *p* Lin. Contrast II < 0.01).

## 4. Discussion

The transformation efficiency of human non-edible biomass into protein food derived from ruminants is relatively low compared to other species such as poultry and swine [18]. However, ruminants are exclusively able to utilize the feed and by-products of either very small or no value for human food as well as non-protein nitrogen to produce high-quality human food, such as milk and beef. Both swine and poultry are not able to digest such raw materials, but in general, they are more efficient in utilizing dietary nutrients. Moreover, their diets can be balanced more precisely. In terms of protein, the ‘ideal protein concept’ allows for the reduction in dietary protein concentration, because the supplementation of individual limiting amino acids then secures both an adequate amount and pattern of amino acids supplied to the intestinal tract [4]. Knowledge on limiting amino acids in ruminants, and especially in beef cattle nutrition under practical feeding conditions, is still scarce. This is mainly due to the difficulty to quantify the amount and pattern of amino acids reaching the small intestine for absorption. Rumen-protected amino acids present an opportunity to overcome the challenge of ruminal degradation. However, to identify a limitation of a certain amino acid, the supplementation of itself to a diet deficient in this amino acid should relieve the animal from the deficiency. Therefore, this results in an increase in growth performance [19]. Numerous studies imply that methionine and lysine may be the most promising amino acids for growth limitation [6]. Therefore, we conducted a feeding trial (published in [6]) to evaluate the role of methionine as a first-limiting amino acid in diets low in crude protein for growing German Simmental bulls. Methionine, however, is not only known as a protein building block via peptide bonds, but also as a functional amino acid that acts as a precursor to numerous metabolites. As such, it plays a pivotal role in epigenetics via DNA methylation [20] and forms a key part in the one-carbon metabolism in its biologically active form of S-adenosyl-methionine. Via the trans-sulfuration pathway and the intermediate metabolite cysteine, it is involved in the synthesis of the antioxidants glutathione and taurine [21]. 

Hence, the objective of this study was to evaluate the effect of RPMET in reduced crude protein diets on metabolic pathways in growing German Simmental bulls under practical feeding conditions. 

As to our knowledge, this is the first study evaluating the metabolic response of German Simmental beef cattle that were fed diets reduced in crude protein supplemented with RPMET. Until 2023, there were 153 publications on metabolomics approaches in research with bovine species available [22]. Recently published studies predominantly aimed to identify biomarkers for desirable economic traits, such as feed efficiency [23,24,25,26,27,28,29,30], growth potential [31] and dairy production [32,33,34]. Metabolomics studies on the effect of supplementing RPMET to dairy/beef diets are limited. Among those few studies, a substantial part focuses on the metabolic programming effect of feeding RPMET to the parental generation. Palombo et al. [35], for example, evaluated the effect of feeding RPMET to late-gestation Holstein dairy cows on metabolic changes in neonatal calves. They found that due to maternal methionine supply neonatal calves experienced beneficial effects on their antioxidant status. The supplementation of RPMET to peripartal dairy cows also led to an improved antioxidant status [21,36,37]. Alfaro et al. [38] conducted a nutrigenetics study in beef cattle. They evaluated the effect of RPMET supplementation on the preconditioning of beef heifers experiencing long-duration transportation stress. They concluded that heifers receiving RPMET had a better-controlled oxidant–antioxidant balance in skeletal muscle. 

Metabolomics is an ‘omics‘ tool to quantify a global set of metabolites within biological samples. Hence, this analysis tool delivers the entirety of metabolic downstream products derived from genomic, transcriptomic and proteomic processes [39]. These datasets reflect the physiological status of cells, and hence enable us to elucidate changes in a biological system induced by different factors, such as environment, diseases and nutrition [22,40,41]. Metabolomics data in combination with phenotype results therefore allow for a more precise understanding of the physiology of the animals [42]. Therefore, we discuss our metabolomics results in context with the performance results of our study [6]. 

In our previous study, bulls were allotted to three different treatment groups: a standard group (CON), which was designed to meet the requirements for German Simmental bulls for fattening at this stage of growth [12] and a diet reduced in crude protein (RED) with supplemental RPMET (RED+RPMET). The latter ones only differed in their digestible methionine concentration (2.2 vs. 3.2 g/kg DM, respectively). As published earlier [6], reduction in dietary crude protein (RED) led to a significant decrease in dry matter intake and hence, metabolizable energy and pre-cecal digestible protein intake as compared to the CON group. The addition of RPMET did not recover feed intake and, therefore, feed and nutrient intake were comparable with the non-supplemented RED group. Consequently, both the bulls from RED and RED+RPMET groups had a lower (*p <* 0.05) growth performance than CON bulls. Metabolomics analyses on a cellular level in muscle, blood and liver tissue revealed that there was hardly any difference in metabolite abundance between CON and both RED and RED+RPMET bulls. This exemplifies the hierarchical concept of nutrient partitioning. The animals set their well-being and maintenance as a prevailing priority of nutrient trafficking (homeostasis) at the expense of (growth) performance (homeorhesis). The lower feed intake in both RED and RED+RPMET led to a lower nutrient availability for metabolism, and hence, bulls grew only to that extent which could be realized with the difference between the total intake of nutrients and energy, and the requirements for maintenance. This concept is controlled at a higher endocrine organ level [43,44] and therefore may have not been visible on a single-cell level as presented via metabolome analysis. 

Considering the actually realized growth rates of both RED and RED+RPMET, the intake of pre-cecal digestible methionine met 101% of RED and 146% of RED+RPMET requirements, respectively. Both RED and RED+RPMET had equal (*p* > 0.05) dry matter and hence, energy and protein intake, which were, from a retrospective point of view, sufficient for their actually realized growth rates. Therefore, we could exclude both energy and protein intake as limiting factors for growth response in the RED+RPMET group. If methionine had been the first-limiting amino acid for growth, dietary protein quality (i.e., amino acid pattern) would have been improved. This means that more amino acids would have been utilized for protein deposition and this would have then resulted in a decrease in fat deposition in RED+RPMET compared to RED. Respective results can be found in [6]. 

However, additional methionine in the RED+RPMET group did not relieve bulls from growth limitation, but altered hepatic anti-oxidant pathways. This may imply that bulls of the RED group used metabolically available methionine for growth as the highest priority, supporting our assumption of nutrient partitioning and the decoupling of homeostasis and homeorhesis. 

Additional methionine in the RED+RPMET group was directed toward anti-oxidant pathways, represented by an increase (*p <* 0.01) in cysteine glutathione disulfide and taurocholic acid, the conjugate of cholic acid and taurine, an important cellular antioxidant defender. The supplementation of RPMET (RED+RPMET) increased (*p* = 0.01) hepatic taurocholic acid concentrations as compared to the non-supplemented group (RED; 4.53 vs. 4.31, respectively) and reached a comparable level to the CON group (4.69). Taurocholic acid, a bile acid, is the conjugate of cholic acid and taurine. Taurine synthesis needs methionine [45] via cysteine (trans-sulfuration pathway) in a three-step enzymatic process. The main biological effects of taurine comprise antioxidant activity by inhibiting mitochondrial reactive oxygen species generation, glucose homeostasis by interfering the insulin-signaling pathway and osmoregulation due to counteracting hyperglycemia-induced osmotic imbalance. 

Hepatic cystine was lower (*p <* 0.01) in both CP-deficient groups (RED and RED+RPMET) as compared to the standard diet (CON). Interestingly, the addition of RPMET (RED+RPMET) failed to increase hepatic cystine concentrations. This would have been reasonable, since cystine is the oxidized form of cysteine, which is directly synthesized from methionine in the trans-sulfuration pathway [8]. It may be that first, other metabolic pathways had higher demands for methionine/methyl groups, or second, that an increase in cystine could not be displayed in that ‘snapshot’ of the metabolism, since it had already been used for other syntheses (e.g., cystine–glutamate antiporter), or third, that it was reduced back to cysteine and as such, used for the synthesis of subsequent metabolites. Glutathione synthesis, for instance, is restricted by the availability of cysteine. Especially in hepatocytes, the trans-sulfuration pathway provides half of the cysteine required for glutathione synthesis, even if cysteine is present in physiological concentrations [46]. Interestingly, the addition of RPMET increased (*p <* 0.01) the abundance of hepatic cysteine glutathione disulfide (4.96 in RED+RPMET vs. 4.63 in RED) and even exceeded the abundance in the standard group (4.79 in CON). 

In conclusion, our results show that growing German Simmental bulls face a dietary protein reduction with nutrient partitioning by setting maintenance and physiological equilibrium as the first priority in nutrient trafficking to ensure that all organs and tissues are interacting correctly. Additional methionine, which was not first limiting for growth under our feeding conditions [6], was directed toward the hepatic synthesis of important anti-oxidant metabolites, such as cysteine glutathione disulfide and taurocholic acid. 

Since our study was the first to elucidate the comprehensive metabolic role of methionine under such feeding conditions, further studies are required to sharpen the role of methionine in growing beef cattle. Further research should evaluate the ‘metabotype’, i.e., the combination of phenotype and metabolome [42], which offers a promising strategy to determine the comprehensive role of amino acids in the metabolism of growing beef cattle.

Knowledge on amino acid partitioning is particularly important to approach the ‘ideal protein concept’ for growing beef cattle. This is pivotal to increase the production efficiency of cattle genetic resources, thereby to drive further bio-economic circularity [2] and improve public health and environmental resilience [3]. 

## Figures and Tables

**Table 1:** Number of features in liver and muscle tissue and blood serum samples of German Simmental bulls for fattening in positive (P) and negative (N) detection modes.

Sample Type	Detection Mode	Number of Features
Liver	P	930
	N	527
Muscle	P	1122
	N	747
Blood serum	P	1835
	N	1379
Total		6540

**Table 2:** Concentration of annotated metabolites in liver and muscle tissue and blood serum samples of growing German Simmental bulls.

						Dietary Treatment				*p*-Value		
						Mean				GLM ^4^	Linear Contrast I ^5^	Linear Contrast II ^6^
Tissue	Mode ^1^	Annotation	Prec m/z*^2^*	Retention Time	Quality ^3^	CON	RED	RED+RPMET	SEM	CON vs. RED vs. RED+RPMET	CON vs. RED+RPMET	RED vs. RED+RPMET
Liver	P	Carnosine	227.11	462.55	4	5.09 ^a^	4.79 ^b^	4.85 ^b^	0.11	<0.01	<0.01	0.31
Liver	P	Cystine	241.03	492	4	4.65 ^a^	4.18 ^b^	4.32 ^b^	0.20	<0.01	<0.01	0.21
Liver	P	Taurocholic acid	516.3	291.72	4	4.69 ^a^	4.31 ^b^	4.53 ^a^	0.15	<0.01	<0.01	0.01
Liver	P	L-leucine/L-isoleucine/norleucine	132.1	318.27	4	4.37 ^a^	4.13 ^b^	4.05 ^b^	0.15	<0.01	<0.01	0.32
Liver	N	Cysteine glutathione disulfide	425.08	512.25	4	4.79 ^b^	4.63 ^c^	4.96 ^a^	0.15	<0.01	0.99	<0.01
Blood serum	N	Pyrrolidone-carboxylic acid	128.04	119.04	4	4.61 ^a^	3.97 ^b^	4.11 ^b^	0.19	<0.01	<0.01	0.14

^1^ Mode describes detection mode with P = positive mode and N = negative mode. ^2^ m/z of precursor. ^3^ Quality denotes the quality of annotation as described in Section 2.3. ^4^ GLM is the general linear model with a Student–Newman–Keuls’ post-hoc test to detect group mean differences. ^5^ *p*-Values of the linear contrast analysis of CON vs. RED+RPMET. ^6^ *p*-Values of the linear contrast analysis of RED vs. RED+RPMET. ^a,b,c^ Values within a row with different superscripts were statistically different in the SNK. Statistical significance was declared at *p* < 0.05.

## Data Availability

We confirm that all data supporting our conclusions are available in the article and accessible in MassIVE MSV000092367.
